# Impact of the Pandemic on Selected Aspects of Health-Promoting Attitudes in 2020–2021: A Cross-Sectional Study

**DOI:** 10.3389/fpubh.2022.916972

**Published:** 2022-07-07

**Authors:** Agnieszka Kułak-Bejda, Grzegorz Bejda, Wojciech Kułak, Andrzej Guzowski, Joanna Fiłon, Cecylia Łukaszuk, Mateusz Cybulski, Napoleon Waszkiewicz, Elzbieta Krajewska-Kułak

**Affiliations:** ^1^Department of Psychiatry, Medical University of Białystok, Białystok, Poland; ^2^The School of Medical Science in Białystok, Białystok, Poland; ^3^Department of Pediatric Rehabilitation and Center of Early Support for Handicapped Children “Give a Chance”, Medical University of Białystok, Białystok, Poland; ^4^Department of Integrated Medical Care, Medical University of Bialystok, Białystok, Poland

**Keywords:** pandemic, urban residents, health-promoting behaviors, COVID-19, impact

## Abstract

**Introduction:**

In the face of the COVID-19 pandemic, people began to change both their health-promoting and anti-health behaviors.

**Aim of the Paper:**

To assess the impact of the pandemic on selected health-promoting attitudes.

**Methods:**

The cross-sectional study was conducted from March 2020 to September 2021. We have used the author's survey questionnaire and the standardized Wellness Behaviors Inventory (WBI). The questionnaires were given to respondents in paper versions to fill it.

**Results:**

The study group included 600 urban residents aged 32–73. Based on the opinions of the respondents, during the pandemic, the following activities increased the most: hand washing (93.3%), eating sweets and snacks (80%), and surfing the Internet (60%). An increase in drug/legal use was reported by 13.3%, with no indication of a decrease or no change in consumption of the above. The overall WBI index for all subjects before the pandemic was 81.3 ± 20.2 points, and the increase significantly (*p* < 0.001) during the pandemic was 87.7 ± 16.7 points. In addition, an increase in preferred eating habits was found (from 19.5 ± 6.4 to 21.1 ± 6.9 points; *p* < 0.001), preferred prophylactic behaviors (from 21.1 ± 6.0 to 22.7 ± 5.2 points; *p* < 0.001) and level of presented health practices during the pandemic (from 20.3 ± 5.1 to 24.7 ± 2.7 points; *p* < 0.001), and a decrease significantly (*p* < 0.001) in the degree of positive mental attitude (from 20.3 ± 5.4 points to 19.3 ± 4.9 points).

**Conclusions:**

Respondents generally rated their own and their family's health as worse during the pandemic period, and this trend continued when broken down by gender, cohabitant, place of residence, and education. According to the largest group of respondents, the frequency of handwashing, eating sweets and snacks, surfing the Internet, and using drugs/legal highs increased the most during the pandemic. The overall WBI index for all respondents before and during the pandemic was slightly higher during the pandemic period. Monitoring health behavior during a pandemic is essential for prevention and health care institutions. Further studies are needed to assess the long-term impact of the pandemic on pro-and anti-health behavior of people.

## Introduction

The concepts of health and disease, which are closely intertwined, have been of interest to humans since the beginning of our civilization, and their definition has been evolving over the centuries. In 1948, the World Health Organization (WHO), adopted a definition that stated that health is “a state of complete physical, mental and social wellbeing and not merely the absence of disease or infirmity^1^”. This definition has been subjected to various modifications over the years, which broadened the scope of health and emphasized the role of its determinants, which led to an interdisciplinary perception of health and the involvement of humanists in cooperation with the medical community (1). The concept of health can also be determined in disciplinary (various approaches of different scientific disciplines to health issues), historical (concerning the development of sciences), and cultural terms (criteria of health, disease, and prevention, as well as the values given to human health in different cultures) (2). The development of medicine, a biomedical approach to health and disease, was formed, which defines health as a physiological and biological state of the body that ensures its proper functioning as a biological whole (2). Health can also be conceptualized as the body's state of equilibrium (homeostasis), in which it functions at an optimal level, and disease occurs when this equilibrium is disturbed, usually under the influence of a disease factor (3). The concept of health can also be considered in three dimensions: the ability to perform the activities of daily living, mental wellbeing, and adherence to health-promoting behaviors (4). Talcott Parsons (5), the creator of the sociological and functionalist concept of health, defines health to be “the state of optimum capacity of an individual for the effective performance of the roles and tasks for which he has been socialized” and disease is a state in which an individual cannot fulfill his/her social roles. Another concept of understanding health is holism, which is a view that denotes holistically explaining various phenomena, including all spheres of human life (biological, mental, social, spiritual) and focusing not only on the biological sphere of man but also on the psychological, social and cultural contexts, which determine health and disease to a greater or lesser extent (6).

The literature (7–9) distinguishes four dimensions of health: biological, psychological, social, and spiritual, and two perspectives of it: objective (medical, psychological) and subjective (from the patient's point of view), the variability of health is emphasized (human life is a process of continuous efforts and changes), the positive understanding of health is stressed (as a potential/resource), the concept of health is identified with the concepts of happiness, wellbeing and quality of life (a sense of happiness is a symptom of health, and health is an essential condition of happiness), and the principle 'your health in your hands' is promoted, whereby everyone is held accountable for their health.

Any behavior that affects a person's health status, either positively or negatively, is considered to be a health-related behavior. Daily habits involving diet, exercise, safety practices, and substance use are not only related to the prevention of disease but also affect the management of chronic illness and degree of disability (10). Common health-related behaviors include diet, exercise, smoking, alcohol use, safety practices, and participation in health screening examinations such as testing for cholesterol levels, and breast and prostate cancer (11).

Regular physical activity is associated with lower death rates for adults and decreases the risk of death from heart disease, lowers the risk of developing diabetes, and helps reduce blood pressure.

The study aimed to assess the impact of the pandemic on selected health-promoting attitudes. We established two research hypotheses. The first one was that the threat related to the risk of Infection with an unknown and dangerous pathogen and a complete change in everyday functioning contributed to positive health-promoting behaviors. The second one was that the pandemic influenced the feeling of deterioration in health.

## Materials and Methods

The permission of the Bioethics Committee APK.002.184.2021 was obtained for conducting the research. The cross-sectional study was conducted from March 2020 to September 2021. A total of 730 questionnaires in a paper version were distributed. In return, we received 600 fully completed questionnaires and qualified for the study. The conditions for entering the study included residence in the city and completion of an anonymous survey.

The study was conducted using the author's survey questionnaire and the standardized Wellness Behaviors Inventory (WBI). The author's survey was initially conducted on a group of 100 people to check the clarity/intelligibility of the questions. The respondents' comments were introduced into the final version of the survey.The WBI questionnaire contained 24 statements describing various health-related behaviors (eating habits, prophylactic behaviors, positive mental attitudes, health practices). The respondent indicated how often they performed a given health-related activity by rating each of the listed behaviors on a five-point scale: (1) hardly ever, (2) rarely, (3) occasionally, (4) often, (5) almost always. Because of the possibility of periodic preference for certain types of health behaviors, it was assumed that the recent year should only be considered in the evaluation. The numerical values marked by the respondent were counted to obtain a range of 24–120 points. The higher the score obtained by the respondent, the higher the intensity of health behaviors he or she declared. When converted to standardized units based on the table below, the overall index was subject to interpretation according to the properties that characterize the sten score. Results within: 1–4 sten were treated as low scores; 7–10 sten—as high; 5 and 6 sten—as average (12).

The internal concordance of the WBI, as determined by Cronbach's *alpha*, is 0.85 for the entire Inventory and ranges from 0.60 to 0.65 for its four subscales. Chi^2^-test with Yate's correction was conducted.

## Results

The cross-sectional study was conducted on the group of 600 urban residents (73.3% residents of cities with a population of 200,000–500,000; 13.3% with a population of 50,000–100,000; and 6.7% with a population of up to 50,000 or more than 500,000). There were 69.2% females in the study group, with a mean age of 58.9 ± 11.9, and 30.8% males aged 57.6 ± 12.1. In general, the age of the respondents ranged from 32 to 73. The largest number of people lived with their spouse, 53.3%. Twenty-eight percent of the respondents lived with a spouse and children, 12% were single, and 6.7% lived with children only. 66.7% of the respondents had higher education, and 33.3% – had secondary education.

In the first part of the survey, respondents were asked to rate their health before and during the pandemic. In general, respondents rated them as worse during the pandemic period. This trend continued when broken down by gender, co-residence, place of residence, and education. The results are illustrated in Table 1.

**Table 1:** Respondents' assessment of their health status – before and during the pandemic.

	**Before the pandemic**					**During the pandemic**				
**Opinion**	**Total** ***N*** **= 600**		**Female** ***N*** **= 415**		**Male** ***N*** **= 185**	**Total** ***N*** **= 600**		**Female** ***N*** **= 415**		**Male** ***N*** **= 185**
**Gender**										
Better	482		330		152	0		0^***^		0^***^
No changes	117		84		33	95		63		32
Worse	1		1		185	416		263^***^		153
**Opinion**	**Total** ***N*** **= 600**	**Single** ***N*** **= 72**	**With children** ***N*** **= 40**	**With spouse** ***N*** **= 320**	**With spouse and children** ***N*** **= 168**	**Total** ***N*** **= 600**	**Single** ***N*** **= 72**	**With children** ***N*** **= 40**	**With spouse** ***N*** **= 320**	**With spouse** **and children** ***N*** **= 168**
**Co-residents**										
Better	482	68	28	262	124	1	0^***^	0	1^*^	0^***^
No changes	117	4	12	57	44	118	2	12	60	44
Worse	1	0	0	1	0	481	70^***^	28^***^	259^***^	124^***^
**Opinion**	**Total** ***N*** **= 600**	**Up to 50,000** ***N*** **= 40**	**50,000 - 100,000** ***N*** **= 80**	**200,000 - 500,000** ***N*** **= 440**	**> 500,000** ***N*** **= 40**	**Total** ***N*** **= 600**	**Up to 50,000** ***N*** **= 40**	**50,000 - 100,000** ***N*** **= 80**	**200,000 - 500,000** ***N*** **= 440**	**> 500,000** ***N*** **= 40**
**City of residence**										
Better	482	36	52	358	36	1	0^***^	0^***^	1^***^	0
No changes	117	4	28	81	4	118	4	28	82	4
Worse	1	0	0	1	0	481	36^***^	52^***^	357^***^	36^***^
**Opinion**	**Total** ***N*** **= 600**		**Secondary** ***N*** **= 200**		**Higher** ***N*** **= 400**	**Total** ***N*** **= 600**		**Secondary** ***N*** **= 200**		**Higher** ***N*** **= 400**
**Education**										
Better	482		174		308	1		0^***^		1^***^
No Changes	117		19		98	118		19		99
Worse	1		0		1	481		174^***^		307^***^

* *p < 0.05.*

*** *p < 0.001 before vs. during the COVID-19 pandemic Chi^2^-test with Yate's correction*.

The respondents were then asked to rate their family's health status before and during the pandemic. In general, the respondents rated their family's health, as in the case of their own health, to be worse during the pandemic. This trend continued, as it did in the case of their own health, when broken down by gender, co-residence, place of residence, and education. The results are illustrated by Table 2.

**Table 2:** Respondents' assessment of the health status of their families – before and during the pandemic.

	**Before the pandemic** ***N*** **= 600**					**During the pandemic** ***N*** **= 600**				
**Opinion**	**Total** ***N*** **= 600**		**Female** ***N*** **= 415**		**Male** ***N*** **= 185**	**Total** ***N*** **= 600**		**Female** ***N*** **= 415**		**Male** ***N*** **= 185**
**Gender**										
Better	387		255		132	0		0^***^		0^***^
No changes	209		156		56	144		104^***^		40
Worse	4		4		0	456		311^***^		145^***^
**Opinion**	**Total** ***N*** **= 600**	**Single** ***N*** **= 72**	**With children** ***N*** **= 40**	**With spouse** ***N*** **= 320**	**With spouse** **and children** ***N*** **= 168**	**Total** ***N*** **= 600**	**Single** ***N*** **= 72**	**With children** ***N*** **= 40**	**With spouse** ***N*** **= 320**	**With spouse** **and children** ***N*** **= 168**
**Whom the respondents live with**										
Better	387	61	22	205	99	0	0^***^	0^***^	0^***^	0^***^
No changes	209	10	18	113	68	144	5	14	76^*^	49
Worse	4	1	0	2	1	456	67^***^	26^*^	244^***^	119^***^
**Opinion**	**Total** ***N*** **= 600**	**Up to 50,000** ***N*** **= 40**	**50,000–100,000** ***N*** **= 80**	**200,000–500,000** ***N*** **= 440**	**> 500,000** ***N*** **= 40**	**Total** ***N*** **= 600**	**Up to 50,000** ***N*** **= 40**	**50,000–100,000** ***N*** **= 80**	**200,000–500,000** ***N*** **= 440**	**> 500,000** ***N*** **= 40**
**City of residence**										
Better	387	29	43	286	29	0	0^***^	0^***^	0^***^	0^***^
No changes	209	11	36	151	11	144	5	31	104^**^	4
Worse	4	0	1	3	0	456	35^***^	49^***^	336^***^	36^***^
**Opinion**	**Total** ***N*** **= 600**		**Secondary** ***N*** **= 200**		**Higher** ***N*** **= 400**	**Total** ***N*** **= 600**		**Secondary** ***N*** **= 200**		**Higher** ***N*** **= 400**
**Education**										
Better	387		174		308	0		0^***^		1^***^
No changes	209		19		98	144		19		99
Worse	4		0		1	456		174^***^		307^***^

* *p < 0.05*.

*** *p < 0.001 before vs. during the COVID−19 pandemic Chi^2^-test with Yate's correction*.

According to the opinions of the majority of respondents, the following activities increased the most: hand washing (93.3%), eating sweets and snacks (80%), and surfing the Internet (60%). However, the consumption of sweetened soft drinks (73.3%), consumption of cereal products (66.7%), consumption of fish, dairy products, and eggs, unsweetened beverages, and drinking coffee (60% each) did not change according to the respondents. The remaining indications are presented in Table 3.

**Table 3:** Respondents' views on changes in health-promoting and anti-health behavior during the pandemic.

**Type of health-promoting/anti-health behavior**	**Opinion (number of people)**			
	**Does not concern me**	**Decrease**	**No changed**	**Increased**
Smoking	476	72	40	12
Drinking alcohol	400	40	40	120
Use of drugs/legal highs	520	0	0	80
Regular physical exercise	40	392	40	128
Walking	0	312	200	88
Cycling	280	152	120	48
Eating regularly	0	152	280	168
Consumption of sweets and snacks (e.g. sugar, honey, chocolates, cookies etc.)	0	80	40	480
Consumption of fat (oil, butter, margarine, cream, sour cream, mayonnaise, etc.)	0	160	200	240
Eating fruit (fruit, kiwi, citrus fruit, berries, dried fruit, etc.)	0	112	200	288
Eating vegetables and grains (vegetables, leafy green vegetables, seeds, nuts etc.)	0	112	280	208
Fish consumption	0	192	360	48
Consumption of meat products (sausages, cold cuts, red meat, poultry, etc.)	0	160	320	120
Consumption of dairy products and eggs (milk, yogurt, cocoa, cheese, scrambled eggs, etc.)	0	0	360	240
Eating cereal products (wholemeal bread, refined breads, groats, etc.)	0	152	400	48
Eating fast food (e.g., KFC, McDonald's, etc.)	360	80	80	80
Consumption of sweetened soft drinks (fruit nectars, sweetened soft drinks)	0	80	440	80
Consumption of unsweetened soft drinks (100% vegetable juices, vegetable-fruit juices, fruit juices)	0	120	360	120
Drinking coffee	80	40	360	120
Sleeping at least 7–9 h	0	320	160	120
Hand washing	0	40	0	560
Surfing the Internet	0	40	200	360

The overall WBI index for all subjects before the pandemic was 87.3 ± 20.2 points. (Min.−26 points and Max−105 points) and 5.7 ± 2.1 sten (Min.−1; Max.−9), and during the pandemic, 87.7 ± 16.7 points (Min.−46; Max.−114) and 6.2 ± 2.4 sten (Min.−1; Max.−10), – which indicates an average level of health behaviors, slightly higher during the pandemic. In general, low levels of health behavior were presented by 20% of the respondents before the pandemic and by 13.3% during the pandemic. The average level of health behavior was presented by 40% of the respondents before the pandemic and 26.7% during the pandemic, whereas high level of health behavior was presented by 40% of the respondents before the pandemic and 60% during the pandemic period. Detailed data are provided in Table 4.

**Table 4:** The type of health-related behaviors before and during the pandemic presented by all respondents.

**WBI**	**Before the pandemic** ***N*** **= 600**					**During the pandemic** ***N*** **= 600**				
	**Hardly ever**	**rarely**	**Occasionally**	**often**	**Almost always**	**Hardly ever**	**rarely**	**Occasionally**	**often**	**Almost always**
I eat a lot of vegetables and fruits	40	80	200	200	80	40	40^***^	160	240	120^*^
I avoid catching colds	80	40	120	200	160	80	0^***^	40^***^	280^**^	200
I take the advice of persons who express concern about my health seriously	120	40	80	240	120	120	160^***^	120^*^	120^***^	80^*^
I rest enough	80	120	120	120	160	0^***^	40^***^	160^*^	160^*^	240^*^
I limit the consumption of such products as animal fats and sugar	80	160	200	80	80	80	80^***^	200	80	160^***^
I have the phone numbers of emergency services noted down	160	0	80	80	280	120^*^	0	120^*^	40^***^	320
I avoid situations that have a depressing effect on me	40	80	120	280	80	40	0^***^	160^*^	280	120^*^
I avoid overworking	80	200	80	120	120	0^***^	40^***^	80	200^***^	280^***^
I take care of proper nutrition	40	120	120	200	120	40	80^*^	120	160	200^***^
I follow my doctor's instructions based on my health status	40	80	40	280	160	80^***^	0^***^	40	200^**^	280
I try to avoid overly strong emotions, stress and tension	40	40	160	280	80	40	40	160	280	80
I control my weight	80	160	80	40	240	0^***^	80^***^	80	80^***^	360^***^
I avoid eating food with preservatives	0	120	160	160	160	0	120	200	40^***^	240^***^
I report regularly for medical check-ups	40	80	120	160	200	80^***^	80	120	80^***^	200
I have friends and a settled family life	80	80	160	80	200	160^***^	160^***^	120	160^***^	0^***^
I sleep enough	80	80	120	120	200	40^***^	40^***^	120	120	280^*^
I avoid salt and highly salted foods	120	80	160	80	160	120	40^***^	160	80	200
I'm trying to figure out how others avoid diseases	120	40	240	80	120	80^*^	0^***^	200	120^*^	200^***^
I avoid such feelings as anger, fear and depression	40	160	120	200	80	40	120	120	240	80
I cut down on smoking tobacco	80	0	0	120	400	0^***^	0	0	120	480^*^
I eat wholemeal bread	40	120	240	80	120	40	80^*^	200	80	200^***^
I seek to obtain medical information and understand the causes of health and illness	40	80	120	200	160	40	0^***^	0^***^	0^***^	560^***^
I have a positive thinking	80	80	120	200	320	80	80	120	160	160^***^
I avoid excessive physical exertion	40	120	240	200	0	80^***^	40^***^	120^***^	160	200^***^
**All respondents** *N* = 600	81.3 ± 20.2 points (26–105)					87.7 ± 16.7 points (46–114)^###^				
	5.7 ± 2.1 sten (1–9)					6.2 ± 2.4 sten (1–10)^###^				
	Low		120			Low		80^*^		
	Average		240			Average		160^***^		
	High		240			High		360^***^		

* *p < 0.05.*

** *p < 0.01*.

*** *p < 0.001 before vs. during the COVID-19 pandemic Chi^2^-test with Yate's correction*.

### *p < 0.001 before vs. during the COVID-19 pandemic Wilcoxon's rank test*.

Before, as well as during the pandemic, higher rates of health-related behaviors were characterized by males, people with secondary education, people living with children, and residents of cities with populations over 500,000. Detailed results are shown in Table 5.

**Table 5:** Rates of health-related behaviors before and during the pandemic by gender, education, co-residents, and place of residence.

**WBI**	**Before the pandemic** ***N*** **= 600**					**During the pandemic** ***N*** **= 600**					p-value^*^
	**Hardly ever**	**rarely**	**Occasionally**	**often**	**Almost always**	**Hardly ever**	**rarely**	**Occasionally**	**often**	**Almost always**	
**GENDER**											
Females *N* = 415	81 ± 29.9 points					86.3 ± 16.3 points					<0.01
	5.2 ± 2.2 sten					5.8 ± 2.3 sten					<0.001
Males *N* = 185	81.9 ± 21 points					91 ± 17.3 points					<0.001
	6.1 ± 2.7 sten					7.2 ± 2.3 sten					<0.001
**EDUCATION**											
Secondary *N* = 200	90.6 ± 10.4 points					93.2 ± 9.2 points					<0.01
	7.2 ± 1.5 sten					7.4 ± 1.4 sten					NS
Higher *N* = 400	76.6 ± 22.2 points					85 ± 18.9 points					<0.001
	5.5 ± 2.6 sten					6.7 ± 2.5 sten					<0.001
**CO-RESIDENTS**											
Singles *N* = 72	81.3 ± 7.9 points					83.6 ± 4.1 points out of 120					<0.05
	5.8 ± 1.4 sten					6.3 ± 0.5 sten out of 10					<0.01
With spouse and children *N* = 168	82.9 ± 16.3 points					95.3 ± 14.6 points					<0.001
	6 ± 2.5 sten					7.7 ± 1.9 sten					<0.001
With spouse *N* = 320	78.7 ± 24.1 points					83.8 ± 18.8 points					<0.01
	5.9 ± 2.6 sten					6.2 ± 2.6 sten					NS
With children *N* = 40	95.4 ± 0.6 points					95.4 ± 0.6 points					NS
	8 ± 0 sten					8 ± 0 sten					NS
**PLACE OF RESIDENCE**											
City up to 50,000 residents *N* = 40	76.4 ± 0.6 points					82.2 ± 0.5 points					<0.001
	5 ± 0 sten					6.0 ± 0 sten					<0.001
City from 50,000 to 100,000 residents *N* = 80	74.5 ± 7.5 points					72.5 ± 3.5 points					<0.05
	5.0 ± 1.0 sten					4.5 ± 0.5 sten					<0.001
City from 200,000 to 500,000 residents *N* = 440	81.1 ± 22.3 points					89.5 ± 17.5 points					<0.001
	6.1 ± 2.6 sten					7.0 ± 2.3 sten					<0.001
City above 500,000 residents *N* = 400	102.1 ± 0.3 points					105.2 ± 0.4 points					<0.001
	9.0 ± 0 sten					9.0 ± 0 sten					NS

* *Wilcoxon rank test*.

There was a general increase in preferred eating habits in the study group during the pandemic (from 19.5 ± 6.4 points to 21.1 ± 6.9 points). The upward trend continued regardless of gender or co-residence. There was also an increase among people with higher education and residents of cities with up to 50,000 and cities with a population of 200,000 to 500,000. The habits did not change in the group with secondary education and in the groups of inhabitants of cities with 50,000 to 100,000 and over 500,000 residents. The results are illustrated in Table 6.

**Table 6:** Assessment of good eating habits among the respondents before and during the pandemic by gender, education, co-residents, and place of residence.

	**WBI result good eating habits**		***p*** -value^*^
	**Before the pandemic**	**During the pandemic**	
Total *N* = 600	19.5 ± 6.4 points	21.1 ± 6.9 points	<0.001
**Gender**			
Females *N* = 415	19.2 ± 6.2 points	20.4 ± 6.8 points	<0.01
Males *N* = 185	20.2 ± 6.7 points	22.5 ± 7.0 points	<0.01
**Co-residents**			
Singles *N* = 72	20.4 ± 3.2 points	22.1 ± 4.4 points	<0.01
With spouse and children *N* = 168	16.9 ± 8.2 points	20.8 ± 9.6 points	<0.001
With spouse *N* = 320	20.3 ± 5.7 points	20.6 ± 6.0 points	NS
With children (*N* = 40)	23.1 ± 0.2 points	24.6 ± 0.3 points	<0.001
**Education**			
Secondary *N* = 200	21.0 ± 3.9 points	21.0 ± 3.9 points	NS
Higher *N* = 400	18.8 ± 7.2 points	20.8 ± 8.0 points	<0.001
**Place of residence**			
City up to 50,000 residents *N* = 40	23.6 ± 0.2 points	26.4 ± 0.2 points	<0.001
City from 50,000 to 100,000 residents *N* = 80	14.4 ± 2.2 points	14.4 ± 2.2 points	NS
City from 200,000 to 500,000 residents *N* = 440	19.3 ± 6.2 points	21.1 ± 6.8 points	<0.001
City above 500,000 residents *N* = 40	30.6 ± 0.1 points	30.6 ± 0.1 points	NS

* *Test-t.

A general increase in the preferred prophylactic behaviors in the study group during the pandemic was observed (from 21.1 ± 6.0 points to 22.7 ± 5.2 points). The upward trend continued regardless of gender, co-residents, education, and place of residence, except for the residents of cities with a population of up to 50,000 inhabitants and over 500,000 inhabitants - where it did not change, and the group of city residents with the population between 50,000 and 100,000, where it decreased. The results are illustrated in Table 7.

**Table 7:** Assessment of prophylactic behaviors among the respondents before and during the pandemic by gender, education, co-residents, and place of residence.

	**WBI result prophylactic behaviors**		***p*** -value^*^
	**Before the pandemic**	**During the pandemic**	
Total *N* = 600	21.1 ± 6.0 points	22.7 ± 5.2 points	<0.001
**Gender**			
Females *N* = 415	21.0 ± 5.9 points	22.2 ± 5.2 points	<0.01
Males *N* = 185	21.4 ± 6.2 points	23.7 ± 5.2 points	<0.001
**Co-residents**			
Singles *N* = 72	22.9 ± 2.9 points	23.6 ± 2.0 points	<0.01
With spouse and children *N* = 168	23.8 ± 6.1 points	26.2 ± 3.7 points	<0.001
With spouse *N* = 320	19.1 ± 6.2 points	20.6 ± 5.7 points	<0.01
With children *N* = 40	23.4 ± 0.2 points	23.8 ± 0.3 points	NS
**Education**			
Secondary *N* = 200	22.6 ± 2.6 points	23.4 ± 2.3 points	<0.01
Higher *N* = 400	20.4 ± 7 points	22.3 ± 6.2 points	<0.001
**Place of residence**			
City up to 50,000 residents *N* = 40	24.2 ± 0.3 points	24.2 ± 0.3 points	NS
City from 50,000 to 100,000 residents *N* = 80	19.0 ± 4.0 points	17.0 ± 3.0 points	<0.001
City from 200,000 to 500,000 residents *N* = 440	20.6 ± 6.4 points	23.1 ± 5.2 points	<0.001
City above 500,000 residents *N* = 40	28.6 ± 0.2 points	28.6 ± 0.2 points	NS

* *Test-t*.

In general, the degree of positive mental attitude was found to decrease significantly (*p* < 0.001) in the study group during the pandemic (from 20.3 ± 5.4 points to 19.3 ± 4.9 points). A similar trend was observed in females and males; in the case of single persons and those living with a spouse, with no changes in the case of persons living with children or with a spouse and children; in the case of persons with secondary education, with practically no differences in the case of persons with higher education and the case of inhabitants of cities with the population of 50,000–100,000 and over 500,000, with practically no changes in the case of inhabitants of cities with the population of 200,000–500,000 and with the degree of positive mental attitude increasing from 18.0 ± 2.6 points to 20.2 ± 0.3 points in residents of cities up to 50,000 inhabitants. Detailed results are shown in Table 8.

**Table 8:** Assessment of positive mental attitudes before and during the pandemic by gender, education, co-residents and place of residence.

	**WBI result positive mental attitude**		***p*** -value^*^
	**Before the pandemic**	**During the pandemic**	
Total *N* = 600	20.3 ± 5.4 points	19.3 ± 4.9 points	<0.001
**Gender**			
Females *N* = 415	20.1 ± 5.3 points	18.9 ± 5.0 points	<0.001
Males *N* = 185	20.6 ± 5.5 points	20.0 ± 4.8 points	<0.05
**Co-residents**			
Singles *N* = 72	21.3 ± 2.9 points	20.3 ± 0.5 points	<0.01
With spouse and children *N* = 168	20.8 ± 4.4 points	20.7 ± 3.3 points	NS
With spouse *N* = 320	19.0 ± 6.0 points	17.4 ± 5.4 points	<0.001
With children (*N* = 40)	26.2 ± 0.3 points	26.2 ± 0.3 points	NS
**Education**			
Secondary *N* = 200	23.0 ± 2.3 points	21 ± 3.6 points	<0.001
Higher *N* = 400	18.9 ± 5.9 points	18.4 ± 5.3 points	NS
**Place of residence**			
City up to 50,000 residents *N* = 40	18.0 ± 2.6 points	20.2 ± 0.3 points	<0.01
City from 50,000 to 100,000 residents *N* = 80	17.0 ± 3.0 points	13.5 ± 1.5 points	<0.001
City from 200,000 to 500,000 residents *N* = 440	20.5 ± 5.7 points	20.0 ± 5.1 points	NS
City above 500,000 residents *N* = 40	26.2 ± 0.2 points	22.3 ± 0.5 points	<0.001

* *Test – t*.

Health practices in the WBI questionnaire include daily sleep and recreation habits or physical activity. Overall, an increase in the level of health practices presented during the pandemic was shown (from 20.3 ± 5.1 points to 24.7 ± 2.7 points). This trend continued irrespective of gender, education, place of residence, and co-residents, except for the group of people living with children, where no differences in this respect were observed. Detailed data are provided in Table 9.

**Table 9:** Assessment of health practices among the respondents before and during the pandemic by gender, education, co-residents, and place of residence.

	**WBI result health practices**		***p*** -value^*^
	**Before the pandemic**	**During the pandemic**	
Total *N* = 600	20.3 ± 5.1 points	24.7 ± 2.7 points	
**Gender**			
Females *N* = 415	20.5 ± 5.1 points	24.6 ± 2.7 points	
Males *N* = 185	20.0 ± 5.2 points	25.1 ± 2.8 points	
**Co-residents**			
Singles *N* = 72	18.7 ± 2.2 points	20.9 ± 0.9 points	
With spouse and children *N* = 168	20.0 ± 3.6 points	26.5 ± 2.3 points	
With spouse *N* = 320	20.3 ± 6.1 points	24.6 ± 2.5 points	
With children *N* = 40	25.3 ± 0.2 points	25.3 ± 0.2 points	
**Education**			
Secondary *N* = 200	23.6 ± 2.7 points	25.8 ± 0.8 points	
Higher *N* = 400	18.7 ± 5.2 points	24.2 ± 3.2 points	<0.001
**Place of residence**			
City up to 50,000 residents *N* = 40	16.0 ± 0 points	20.0 ± 0 points	
City from 50,000 to 100,000 residents *N* = 80	22.0 ± 2.01 points	26.0 ± 0 points	
City from 200,000 to 500,000 residents *N* = 440	20.6 ± 5.6 points	24.9 ± 2.8 points	
City above 500,000 residents *N* = 40	18.3 ± 0.2 points	25.8 ± 0.4 points	

* *Test – t*.

## Discussion

The current study aimed to assess the impact of the COVD-19 pandemics on selected health-promoting behaviors among adult city residents. The respondents generally rated their own and their family's health as worse during the pandemic. There was also an increase in preferred eating habits, preferred prophylactic behaviors, presented health practices, and a decrease in the degree of positive mental attitude.

The changes that occurred due to the pandemic were multifaceted, as they affected not only the society as a whole but above all, every individual. The sheer risk associated with the danger of Infection with an unknown and dangerous pathogen and a complete change in the scope of everyday functioning could undoubtedly become a reason for changes in health-promoting behavior. They are shaped by many factors, such as conscious choices and lifestyles, and health habits formed during socialization and modified and reinforced in adulthood (13).

The pandemic has impacted Poles started approaching their health. It has been shown that more than half of the respondents admitted that they care about their health more than in the corresponding period of the previous year (including 25% of Poles who “care much more about their health”)^2^. In the present study, the respondents rated their health status, as well as the health of their families, as worse during the pandemic compared to the period before it. This trend continued regardless of gender, co-residents, place of residence, or education.

The respondents took the most often to take care of their health (physical and mental) included getting enough sleep, enough rest, and eating healthy foods (14, 15). The study 'Hygiene habits of Poles during the coronavirus pandemic' commissioned by NAOS in May 2021 on a representative group of Poles (1,025) aged 18–65, who completed an online survey, showed that in the first months of the pandemic, the percentage of persons who washed hands increased to 65% (14).

One aspect of health-promoting behavior includes proper eating habits – primarily the type of consumed food. According to the study by Sidor and Roman (16), 45.3% of subjects consumed more food during the lockdown than the period before the pandemic. Also, the results from the international ECLB-COVID-19 study, conducted *via* an online survey among Asian, European, and African subjects, indicated an increase in consumption of unhealthy snacks, uncontrolled eating, eating between meals, an increased number of consumed meals (14). However, in the present study, the pandemic was conducive to increasing good eating habits.

An essential aspect of health-promoting activities is prophylactic behavior. Wypych-Slusarska et al. (16), in a group of 245 Polish adults, assessed the frequency of consuming selected products and the use of supplementation and prophylactic behaviors related to COVID-19 pandemic. The measures taken by the study participants to boost immunity included vaccinations and vitamin D and C supplementation. In the present study, it was generally found that the number of preferred prophylactic behaviors increased during the pandemic, regardless of gender, co-residents, education, and residence in cities of 200,000–500,000 residents.

Physical activity is another important aspect of health-promoting behavior. In a systematic review related to physical activity during the COVID-19 pandemic (16), Caputo and Reichert (17) found that social isolation affected the decrease in physical activity. The results of Lesser and Nienhuis' study also indicated that physical activity reduced anxiety. Confirmation of the above was also found in the present study. In contrast, in the study by Wypych-Slusarska et al. nearly half of the respondents reported that their level of physical activity did not change during the pandemic. However, when they reopened in the summer of 2020, activity increased by 2 to 62% at that time (18). In a survey conducted in Poland on a representative sample of 1,000 Poles over the age of 18, from 23 to April 30 2020, before the pandemic 65% undertook physical activity at least once a month. During the pandemic, 43% were physically active (19). In the present study, a general increase in the health practices presented during the pandemic. During the pandemic, respondents rested more, avoided overwork, spent more time sleeping, and avoided excessive exercise. This trend continued irrespective of gender, education, place of residence, and co-residents, except for the group of people living with children, where no differences in this respect were observed.

Adopting a positive mindset is conducive to better health and maintaining mental resilience. The Dialogue Therapy Center asked a representative sample of 350 psychiatrists from across the country how they assess the current mental state of Poles. It turned out that 74.3% of the respondents felt it was worse than two years ago, i.e., before the COVID-19 outbreak (20). According to a survey conducted by UCE RESEARCH and SYNO Poland, among 1,040 Poles aged 18–80, 38.5% of respondents believed their mental health deteriorated during the pandemic^3^. The study found that 68% of the respondents who identified mental health deterioration syndromes had not noticed them before the pandemic. The most commonly reported symptoms of impaired mental health were lowered mood, sleep disturbances, impaired concentration and attention, a pessimistic view of the future, and low self-esteem and self-confidence. In the present study, the positive mental attitudes of the respondents were assessed using the WBI questionnaire. The study group showed a decrease in positive mental attitude during the pandemic. A similar trend was observed in females and males, and single persons. Also, education had no impact in positive mental attitude during the pandemic. The number of reposndents who declared 'almost always' when taking tips from people who expressed concern about their health decreased (from 20% pre-pandemic to 13.3% during the pandemic).

In contrast, there was an increase in 'almost always' statements regarding avoiding situations that make the respondents feel depressed (from 13.3% pre-pandemic to 20% during the pandemic). This issue of avoiding overly strong emotions, stress, and tension, and feelings such as anger, anxiety, and depression remained at a similar level (13.3% before and during the pandemic). Interestingly, 33.3% of the respondents declared before the pandemic that they “almost always” had friends and settled family life, while no one thought so during the pandemic. The number of those declaring “almost always” with regard to positive thinking also decreased (from 53.3% before the pandemic to 26.7% during the pandemic).

The Global Drug Survey report, which is based on data collected in May and June 2020 from 58,811 people in Germany, France, the United Kingdom, Ireland, Austria, the Netherlands, Switzerland, Australia, New Zealand, Brazil and the United States, showed that 43% of respondents were more likely to use alcohol during the pandemic, 39% consumed more alcohol, 29% drank less alcohol and 24% reported no change. They cited “more time to drink” as the most common reason for changing their drinking style (42%) and “boredom” (41%). In contrast, the others indicated compensation for anxiety and worries caused by the pandemic (see text footnote 3). In the present survey, an increase in alcohol consumption was declared by 20% of the respondents, compared to 6.7% who said nothing had changed in this area and 6.7% who reported a decrease in alcohol use.

The report lists the top 10 most commonly identified stimulants used by respondents in 2020. Alcohol came in first (94%), followed by cannabis containing THC (64.5%), and tobacco (60.8%). In addition to alcohol and tobacco products, the list contains the following: MDMA, CBD-only (non-psychoactive) cannabis, cocaine, amphetamine, LSD, benzodiazepines, hallucinogenic mushrooms, ketamine, and prescription opioids (see text footnote 3). Lockdown was also found to increase the use of cannabis containing the psychoactive THC. Thirty-nine percent said they smoked more, but another 39% claimed they smoked the same amount, and only 21% said their marijuana use had decreased. However, the above may be related to problems of accessibility to such stimulants during the closure of borders and public places (see text footnote 3).

What may be a cause for concern in our study is that respondents reported an increase in drug/legal highs consumption.

In other studies conducted in different countries during the pandemic of COVId-19, the pandemic had a significant impact on the change of habits and psychological wellbeing of populations in different manners (e.g., smoking, physical activity, eating habits)^4^ (21–28).

This may indicate that the pandemic has had a global impact on health behaviors.

### Limitations of the Study

The study group included a population of only 600 residents, so it cannot be generalized to the entire population of Poland. The study was limited by the lack of inclusion of a rural population group. The study group should be larger and expanded to include such individuals in future studies.

## Conclusions

The respondents rated their own and their families' health as worse during the pandemic. During the pandemic period, handwashing, consumption of sweets and snacks, Internet surfing, and drug/legal high use increased the most. The overall index of WBI for all subjects was slightly higher during the pandemic. An increase in preferred dietary habits, preferred prophylactic behaviors, and presented health practices was noted, whereas the degree of positive mental attitude decreased. Rates of health-related behaviors depended on gender, education, place of residence, and co-residents.

## Data Availability Statement

The raw data supporting the conclusions of this article will be made available by the authors, without undue reservation.

## Ethics Statement

The studies involving human participants were reviewed and approved by the Bioethics Committee of the Medical University of Bialystok APK.002.33.2021. Written informed consent for participation was not required for this study in accordance with the national legislation and the institutional requirements.

## Author Contributions

AK-B, EK-K, and GB: designed the study and wrote the protocol. AK-B, GB, WK, AG, JF, CŁ, MC, NW, and EK-K: data collection. WK: undertook the statistical analysis. AK-B and GB: wrote the first draft of the manuscript. All authors contributed to and have approved the final manuscript.

## Conflict of Interest

The authors declare that the research was conducted in the absence of any commercial or financial relationships that could be construed as a potential conflict of interest.

## Publisher's Note

All claims expressed in this article are solely those of the authors and do not necessarily represent those of their affiliated organizations, or those of the publisher, the editors and the reviewers. Any product that may be evaluated in this article, or claim that may be made by its manufacturer, is not guaranteed or endorsed by the publisher.

## Footnotes

^1^https://www.who.int/governance/eb/who_constitution_en.pdf (accessed April 4, 2022).

^2^https://www.rynekzdrowia.pl/Uslugi-medyczne/Badanie-IBRiS-ponadpolowa-Polakow-dba-o-swoje-zdrowie-bardziej-niz-przed-rokiem,206687,8.html (accessed April 4, 2022).

^3^https://pulsmedycyny.pl/psychiatrzy-coraz-wiecej-polakow-w-gabinetachrosnie-liczba-interwencji-kryzysowych-sondaz-1129775 (accessed April 04, 2022).

^4^https://www.globaldrugsurvey.com/gds-covid-19-special-edition-key-findings-report/ (accessed April 04, 2022).
